# Malignant Catatonia Mimicking Pheochromocytoma

**DOI:** 10.1155/2013/815821

**Published:** 2013-10-22

**Authors:** Sophia Wong, Barbara Hughes, Morris Pudek, Dailin Li

**Affiliations:** ^1^Department of Pathology and Laboratory Medicine, University of British Columbia, 855 West 12th Avenue, Vancouver, BC, Canada V5Z 1M9; ^2^Department of Medicine, Ridge Meadows Hospital, 11666 Laity Street, Maple Ridge, BC, Canada V2X 7G5; ^3^Division of Clinical Chemistry, Department of Pathology and Laboratory Medicine, Vancouver General Hospital, 855 West 12th Avenue, Vancouver, BC, Canada V5Z 1M9

## Abstract

Malignant catatonia is an unusual and highly fatal neuropsychiatric condition which can present with clinical and biochemical manifestations similar to those of pheochromocytoma. Differentiating between the two diseases is essential as management options greatly diverge. We describe a case of malignant catatonia in a 20-year-old male who presented with concurrent psychotic symptoms and autonomic instability, with markedly increased 24-hour urinary levels of norepinephrine at 1752 nmol/day (normal, 89–470 nmol/day), epinephrine at 1045 nmol/day (normal, <160 nmol/day), and dopamine at 7.9 **μ**mol/day (normal, 0.4–3.3 **μ**mol/day). The patient was treated with multiple sessions of electroconvulsive therapy, which led to complete clinical resolution. Repeat urine collections within weeks of this presenting event revealed normalization or near normalization of his catecholamine and metanephrine levels. Malignant catatonia should be considered in the differential diagnosis of the hypercatecholamine state, particularly in a patient who also exhibits concurrent catatonic features.

## 1. Introduction

Malignant catatonia (MC) is an uncommon and highly lethal neuropsychiatric condition first reported by Calmeil in 1832 [1]. Since then, it has been described in the medical literature by a litany of names, among them pernicious catatonia, lethal catatonia, and acute fulminating psychosis [2]. Similar to MC, pheochromocytoma is also a potentially fatal disease, and the two conditions can present with overlapping clinical and biochemical traits. It is vital that these pathologies be differentiated, as therapeutic options greatly diverge between the two. 

## 2. Case Presentation

A 20-year-old male was seen by the internal medicine service for fever and hypertension NYD (not yet diagnosed). The patient initially presented to the hospital for abnormal behavior, hallucinations, and delusions. He had become increasingly withdrawn over the past few months and began to experience auditory and visual hallucinations several days prior to admission. The patient denied any chills or rigors. He had a chronic productive cough and a persistent sore throat but no other infectious symptoms. There was no history of headaches or seizures.

He was started on sertraline 25 mg daily three days before his hospitalization, although his compliance was questionable. He recently completed a full course of amoxicillin and clarithromycin for pharyngitis, but these provided minimal alleviation of his complaints. He was not on any other regular medications at home. The patient was a nonsmoker and had not ingested any alcohol in recent months. He was a chronic cannabis user who had smoked marijuana almost daily for the past three to four years and had also consumed some psilocybin mushrooms two weeks prior to admission. Family history was negative for hypertension, pheochromocytomas, or other endocrine tumors. 

On examination, the patient was noted to be markedly diaphoretic, with flushing over his cheeks. His blood pressure was 157/112 mm Hg, heart rate 124 beats per minute, and temperature 37.8°C. The respiratory rate was 18 breaths per minute and oxygen saturation 98% on room air. He appeared tremulous and was stiff in his movements. He responded to questions with single-word answers and was resistant to most commands. He refused to open his mouth for examination. His neurological examination was grossly intact. Cardiac examination revealed normal S1 and S2 with no extra heart sounds. There was a grade 1/6 systolic murmur heard throughout the precordium. Respiratory, abdominal, and rheumatological examinations were unremarkable. 

Laboratory investigations revealed leukocytosis of 13.9 × 10^9^/L (normal, 4.0–11.0 × 10^9^/L) with absolute neutrophils of 12.1 × 10^9^/L (normal, 2.0–8.0 × 10^9^/L) and absolute monocytes of 1.0 × 10^9^/L (normal, 0.1–0.8 × 10^9^/L). C-reactive protein (CRP) was elevated at 41 mg/L (normal, <10 mg/L). Throat, urine, and blood cultures were negative. A lumbar puncture was performed, with normal cerebrospinal fluid (CSF) analysis and cytology. The CSF culture was unremarkable; testing for syphilis and toxoplasma serology, cryptococcal antigen, enterovirus ribonucleic acid (RNA), herpes simplex virus deoxyribonucleic acid (DNA), and acid fast bacilli smear was all negative. 

Extended electrolytes, glucose, and renal and liver function results returned within normal limits. Ethanol level was <2.0 mmol/L, and urine drug screen detected only the presence of cannabinoids and benzodiazepines. An autoimmune workup, including antinuclear antibodies (ANA), antineutrophil cytoplasmic antibodies (ANCA), autoantibodies to extractable nuclear antigens (ENA), rheumatoid factor (RF), and antithyroperoxidase antibodies (TPOAb), was negative. Creatine kinase was 256 U/L (normal, <165 U/L), thyroid stimulating hormone (TSH) 1.43 mU/L (normal, 0.30–5.50 mU/L), and ceruloplasmin 304 mg/L (normal, 220–495 mg/L). Supine renin level was elevated at 0.40 ng/L/s (normal, <0.28 ng/L/s) but the supine aldosterone level was unremarkable at 182 pmol/L (normal, 30–415 pmol/L). Twenty-four-hour urinary catecholamines revealed strikingly increased excretions of norepinephrine at 1752 nmol/day (normal, 89–470 nmol/day), epinephrine at 1045 nmol/day (normal, <160 nmol/day), and dopamine at 7.9 *μ*mol/day (normal, 0.4–3.3 *μ*mol/day). Urinary metanephrine levels were not obtained. 

Computed tomography (CT) of the head revealed no intracranial abnormality. Electroencephalography was normal and no unusual findings were noted on echocardiography. 

The patient was admitted and his sertraline dose increased to 50 mg daily. He was also prescribed quetiapine (which worsened his agitation), as well as PRN loxapine and lorazepam. Because a diagnosis of serotonin syndrome was considered, the patient's sertraline and quetiapine were subsequently discontinued. He was also prophylactically started on meropenem, but the antibiotic was stopped when all cultures returned negative. For blood pressure and heart rate control, he was initiated on metoprolol 12.5 mg twice daily; this was eventually increased to 25 mg twice daily and supplemented by terazosin 1 mg daily.

Interestingly, the patient presented with near identical symptoms of psychosis and autonomic instability four years ago, following what appeared to be an episode of tonsillitis. He later developed catatonia during his hospitalization. A diagnosis of MC was made, and he was treated with multiple sessions of electroconvulsive therapy (ECT). Due to the similarity of the patient's current presentation to this previous event, a trial of ECT was introduced, with striking improvement in his clinical status. The patient required a total of nine ECT sessions over a three-week period for complete resolution of his psychiatric features and autonomic instability.

Eight days after the patient was started on ECT for presumed recurrent MC, a repeat 24-hour urinary specimen was collected to confirm prior results. This was performed while the patient was on metoprolol and terazosin. The urinary catecholamines showed much improved, albeit mildly elevated, norepinephrine of 524 nmol/day and epinephrine of 308 nmol/day. Dopamine, metanephrine, and normetanephrine excretions were normal at 3.0 *μ*mol/day, 1.58 *μ*mol/day (normal, 0.26–1.73 *μ*mol/day), and 1.66 *μ*mol/day (normal, 0.48–2.42 *μ*mol/day), respectively. Another urine collection twelve days thereafter revealed norepinephrine, epinephrine, and dopamine levels of 550 nmol/day, 98 nmol/day, and 2.2 *μ*mol/day, respectively. Metanephrine and normetanephrine excretions were within reference limits at 0.91 *μ*mol/day and 2.29 *μ*mol/day, respectively. As the patient's urinary catecholamine and metanephrine levels normalized or near normalized with treatment of his psychiatric illness, no further investigations, such as an abdominal/pelvic CT, were performed. His initial markedly elevated urinary catecholamine results were thought to be secondary to MC.

## 3. Discussion

Similar to simple or nonmalignant catatonia, patients with MC exhibit the following clinical symptoms and signs [3]:

1. psychosocial withdrawal (mutism, stupor, staring, and negativism) and/or hyperactivity (impulsivity, combativeness, and nudism);
2. motor features (posturing, rigidity, and waxy flexibility);
3. bizarre repetitious behaviors (mannerism, stereotypy, echophenomena, and command automatism).

At least two of the above features, lasting for a minimum of several to twenty-four hours, are needed for a diagnosis of catatonia [2]. Rating scales, such as the Bush-Francis catatonia rating scale [4], the Bräunig catatonia rating scale [5], and the Northoff catatonia scale [6], may assist in its recognition. When individuals with catatonia develop hyperthermia and/or autonomic instability, such as tachycardia, labile/elevated blood pressure, tachypnea, diaphoresis, urinary retention/incontinence, constipation, and/or acrocyanosis, their catatonia has evolved into the “malignant” type, and rapid deterioration of their clinical status often ensues [3, 7, 8]. 

In contrast, pheochromocytoma is a rare catecholamine-secreting tumor with an incidence of <0.3% in hypertensive patients [9] and up to 4.2% in patients with an adrenal incidentaloma [10]. The classic triad of episodic headache, sweating, and tachycardia is only present in 24% of cases [11]. Hypertension may be sustained and/or paroxysmal; some patients may even be normotensive, especially if the tumor is discovered incidentally on imaging, if screening is performed for familial cases, or if the tumor is secreting solely dopamine [12]. Other clinical features associated with pheochromocytoma, particularly those which overlap with symptoms of MC, include flushing/warmth, hyperthermia, heat intolerance, tremulousness, anxiety, dyspnea, and constipation [9, 12]. Screening for pheochromocytoma is usually achieved via 24-hour urinary total/fractionated metanephrines and/or catecholamines or plasma fractionated metanephrines [13]. Markedly elevated results (defined as greater than two or three times the upper limit of normal) warrant further investigations [14]. Once the diagnosis is confirmed biochemically, various imaging modalities, including CT, magnetic resonance imaging (MRI), iodine-123-labelled metaiodobenzylguanidine (^123^I-MIBG) scintigraphy, fluorodeoxyglucose positron emission tomography (FDG-PET), and indium-111-pentetreotide scintigraphy (Octreoscan), may be used to elucidate the location of the tumor [15]. Most pheochromocytomas are curable by surgical resection; for malignant cases, tumor mass reduction, along with radionuclide therapy with or without chemotherapy, is the mainstay of treatment [15].

In the preneuroleptic era, MC comprised 0.25–3.5% of admissions to the psychiatric ward and had a fatal outcome in 75–100% of cases. Its prevalence and lethality have diminished to 0.13–0.50% and 60%, respectively, in the postneuroleptic period [16]. The mean age of onset of MC is 33 years, with a female preponderance (male to female ratio of 1 : 2) [16]. Association with psychosis is most common; however, mood disorders, neurological and medical precipitants (including infectious, metabolic, and/or toxic derangements), medications, and idiopathic causes have also been linked to MC [3]. 

MC consists of three stages. Patients first experience a prodrome of anorexia, insomnia, and mood lability, lasting on average of two weeks in duration. This is then followed by a period of relentless motor agitation, often with violent aggression, auditory and visual hallucinations, and bizarre delusions. Patients also demonstrate catatonic signs and autonomic instability and may refuse all oral intake, leading to dehydration and electrolyte imbalances [16]. This second phase may last for hours to weeks. The final stage is depicted by severe hyperpyrexia and stuporous exhaustion, ending in cardiovascular collapse, coma, and death [16]. 

Although the pathophysiology of MC is not well defined, central dopaminergic hypoactivity, particularly in the basal ganglia-thalamocortical circuits, has been proposed as the underlying mechanism [3, 17]. Alterations in norepinephrine, serotonin, gamma-aminobutyric acid (GABA), and glutamate neurotransmission may also contribute to the development of MC [3, 18]. The clinical features of MC may seem similar to those of neuroleptic malignant syndrome (NMS) and serotonin syndrome. In fact, both conditions are now regarded as iatrogenic variants of MC [8, 19–21], triggered by the use of antipsychotic or antidepressant agents, respectively.

Laboratory findings in MC are nonspecific and include leukocytosis [22], elevated creatine kinase [23], decreased serum iron [24], and increased transaminases [17]. Because the patient's clinical presentation resembles that of an infectious encephalopathy, an extensive neurological workup is often undertaken but yields no significant findings [21]. In our patient, 24-hour urinary catecholamine levels were markedly elevated, with norepinephrine excretion greater than 3.5 times the upper limit of normal (ULN), epinephrine excretion greater than 6.5 times ULN, and dopamine excretion greater than 2.3 times ULN. These results are in accordance with the increased urinary catecholamine and metanephrine values described in other case reports for both MC and NMS [25–27]. 

It is important to note that quetiapine, an atypical antipsychotic with alpha-adrenergic antagonism and norepinephrine reuptake inhibition properties, has been described in the literature to raise normetanephrine (and to a lesser extent, metanephrine) results [28, 29], with presumed effects on catecholamine levels as well. Our patient only received two low doses of quetiapine (50–100 mg) prior to sample collection, and such a striking elevation in urinary catecholamines secondary to this seemed unlikely. Further, on both repeat urinary specimens, the catecholamine and metanephrine values were either within, or mildly above, normal limits, despite the patient now being on two medications (an alpha-blocker and a beta-blocker) that are known to increase urine catecholamine and/or metanephrine levels [29–31]. These results on repeat testing are reassuring in ruling out the presence of a pheochromocytoma and support the clinician's decision to forego unwarranted imaging that would have exposed the patient to a considerable dose of ionizing radiation [32].

Regardless of its initial etiology, fully-developed MC is frequently lethal and necessitates urgent intervention. Aggressive supportive care, such as intravenous fluids and cooling devices, should be instituted early. Blood pressure and cardiac rhythm should be closely monitored and managed appropriately. Conventional and atypical neuroleptics are generally ineffective in the treatment of MC and may in fact aggravate the condition once it is in the advanced stages [33, 34]. Benzodiazepines (e.g., high-dose lorazepam at 8–16 mg/day) [35] are frequently prescribed and can improve the clinical course in most patients [3], but have limited use once MC becomes fulminant [33]. ECT is the most definitive and effective therapeutic option currently available, especially for patients refractory to benzodiazepines and/or those with MC secondary to a functional psychiatric cause [16]. For MC associated with an organic etiology, treatment should be aimed at the underlying disorder. ECT may, nevertheless, provide some relief of MC symptoms, although any effects achieved are likely temporary if the primary process remains uncorrected [16]. 

In summary, we present a case of MC with clinical and biochemical findings mimicking those seen in pheochromocytoma. Treatment of the patient's MC resulted in complete resolution of his hyperadrenergic symptoms and normalization or near normalization of his urinary catecholamine and metanephrine levels. MC should be considered in the differential diagnosis of the hypercatecholamine state, especially in a patient who also exhibits concurrent catatonic features. Although rare, a missed diagnosis of MC may have grave consequences. 
